# Retinal Microvasculature and Visual Acuity after Intravitreal Aflibercept in Diabetic Macular Edema: An Optical Coherence Tomography Angiography Study

**DOI:** 10.1038/s41598-018-38248-1

**Published:** 2019-02-07

**Authors:** Caleb Busch, Taku Wakabayashi, Tatsuhiko Sato, Yoko Fukushima, Chikako Hara, Nobuhiko Shiraki, Andrew Winegarner, Kentaro Nishida, Hirokazu Sakaguchi, Kohji Nishida

**Affiliations:** Department of Ophthalmology, Osaka University Graduate School of Medicine, Suita, Japan

## Abstract

We investigated changes in retinal vascular area and the foveal avascular zone (FAZ) after intravitreal aflibercept in diabetic macular edema (DME) and the association of these changes with visual outcomes. The retinal vascular area in the superficial capillary plexus (SCP) and the deep capillary plexus (DCP) and the FAZ area were measured using optical coherence tomography angiography (OCTA) in 23 eyes of 23 patients with DME, before and after intravitreal aflibercept. Overall, there was no significant change in retinal vascular area or FAZ. Better BCVA after treatment was significantly associated with larger retinal vascular area in the SCP and the DCP, both at baseline (R^2^ = 0.512, P < 0.001 and R^2^ = 0.361, P = 0.002, respectively) and after intravitreal aflibercept (R^2^ = 0.717, P < 0.001 and R^2^ = 0.618, P < 0.001, respectively). MAs were observed in the DCP in 20 eyes (87%), but only detected in four eyes (17%) in the SCP before treatment. The number of eyes with MAs in the DCP significantly decreased to 13 (57%) after treatment (P = 0.049). The persistence of DME was associated with persistent MAs (P = 0.019) and less visual gain (P = 0.031) following treatment. Thus, preserving retinal perfusion and the resolution of MAs are associated with better vision and resolution of the DME after intravitreal aflibercept.

## Introduction

Diabetic retinopathy (DR) is a major complication of diabetes and a leading cause of blindness among the working-age population worldwide^1^. DR is characterized by increased vascular permeability due to the breakdown of the blood-retinal barrier (BRB), or by microaneurysm (MA) formation, capillary nonperfusion, and neovascularization within the retina^2–5^. Diabetic macular edema (DME) is a consequence of DR in the macula, defined as retinal thickening resulting from the leakage from retinal capillaries into the retinal tissue^6,7^. The vascular endothelial growth factor (VEGF) has been shown to play a central role in the pathogenesis of DME. Inhibition of VEGF with anti-VEGF agents such as ranibizumab, bevacizumab, and aflibercept has been effective in treating macular edema and improving vision in DME^7–11^. However, the visual outcomes vary in patients with DME^11^. Previous reports have shown that retinal perfusion evaluated by fluorescein angiography (FA) predicts visual outcomes following anti-VEGF therapy for DME. The visual improvement after treatment correlates with less ischemia and better perfusion in the retina^12^. Therefore, a detailed evaluation of the influence on retinal perfusion following anti-VEGF therapy is needed, to achieve better long-term visual outcomes in DME. Although FA is the gold-standard for evaluating retinal perfusion, inaccurate microvasculature images due to fluorescein dye leakage and an inability to evaluate the deep capillary plexus (DCP) have limited our understanding of microvascular changes that may occur in response to anti-VEGF therapy in DME^13^.

Optical coherence tomography angiography (OCTA) is a novel imaging modality that is able to rapidly and noninvasively visualize retinal blood flow^14–16^. OCTA can visualize both superficial and deep capillary plexuses without the use of exogenous dyes. Recent studies have validated that OCTA can objectively measure retinal capillary density in both healthy and diseased eyes, including in patients with DR^17–21^. A potential association of retinal vascular density (VD) with visual acuity in DR has been reported^22–24^. Changes in the VD and the FAZ area following anti-VEGF therapy in DME have also been reported^25^. However, changes in the microvasculature following anti-VEGF therapy and their association with visual changes have not yet been fully elucidated.

In this study, we assessed the retinal vascular area and the FAZ before and after intravitreal aflibercept injections for DME, and assess the relationship between changes in these measures and visual outcomes.

## Results

Thirty eyes who were treated with aflibercept therapy for DME were initially enrolled. Seven eyes were subsequently excluded because of significant auto-segmentation error (5 eyes) and motion artifact (2 eyes). Therefore, 23 eyes met the criteria for data analysis. Baseline characteristics are shown in Table 1. The patient age was 64.3 ± 11.9 (range, 43–84) years. The baseline logMAR BCVA before treatment was 0.28 ± 0.23. The mean time between the initial intravitreal aflibercept injection and OCTA examination after aflibercept was 8.5 ± 5.6 (range: 3–25) months, and patients received an average of 2.6 ± 1.3 (1–8) aflibercept injections. At the time of the OCTA examination, the mean logMAR BCVA significantly improved to 0.15 ± 0.22 (P = 0.003). The central retinal thickness significantly decreased from 484 ± 133 (313–828) μm to 275 ± 62 (190–458) μm after treatment (*P* < 0.001).

**Table 1:** Patient characteristics.

Eyes/patients, no.	23/23
Age (years) (mean ± SD; range)	64.3 ± 11.9 (43–84)
Sex, no. (%)	
Men	17 (74)
Women	6 (26)
Eye, no. (%)	
Right	11 (48)
Left	12 (52)
Diabetes, no. (%)	
NPDR	14 (61)
PDR	9 (39)
BCVA before treatment	
Landolt C acuity chart (mean; range)	0.52 (0.15–1.0)
LogMAR (mean ± SD)	0.28 ± 0.23
CRT before treatment (µm) (mean ± SD; range)	484 ± 133 (313–828)
Duration of follow-up (months) (mean ± SD; range)*	8.5 ± 5.6 (3–25)
Number of injections during follow-up, no. (mean ± SD; range)	2.6 ± 1.3 (1–8)

SD, standard deviation; BCVA, best-corrected visual acuity; logMAR, logarithm of the minimum angle of resolution; CRT, central retinal thickness.

*Follow-up duration between the initial optical coherence tomography angiography (OCTA) examination before treatment and the OCTA examination after treatment for diabetic macular edema.

### OCTA Findings Before and After Intravitreal Aflibercept

**Figure 1:**
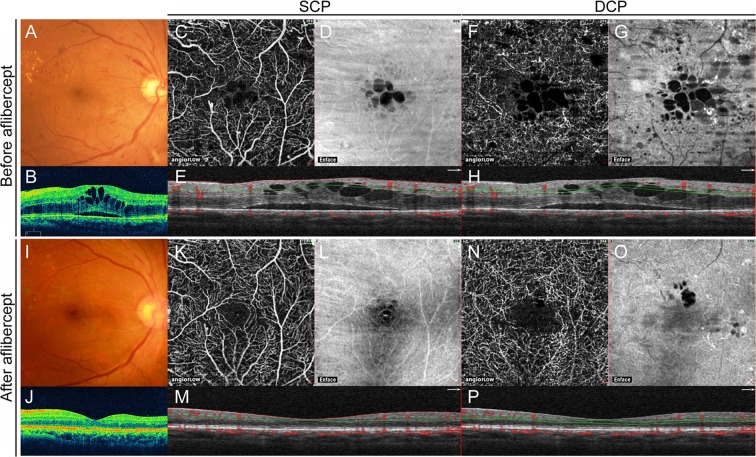
Retinal images obtained from a 43-year-old woman before and after intravitreal aflibercept therapy for diabetic macular edema (DME). (**A**) Fundus photograph obtained before treatment. Visual acuity was 20/40. (**B**) Horizontally oriented spectral-domain optical coherence tomography (SD-OCT) images before treatment. Macular edema involving the fovea is apparent and central retinal thickness (CRT) is 628 µm. (**C**) Optical coherence tomography angiography (OCTA) images of the superficial capillary plexus (SCP) obtained before treatment. (**D**) The enface image. (**E**) The corresponding OCT cross-sectional image through the fovea is shown directly below. The retinal vascular area is 3.82 mm^2^. (**F**) OCTA images of the deep DCP obtained before treatment. (**G**) The enface image. (**H**) The corresponding OCT cross-sectional image through the fovea is shown directly below. The retinal vascular area is 4.06 mm^2^. (**I**) Fundus photograph obtained 12 months after treatment. The patient received four intravitreal aflibercept injections. Visual acuity was 20/22. (**J**) Horizontally oriented spectral-domain optical coherence tomography (SD-OCT) images after treatment. Macular edema is resolved, and CRT is 276 µm. (**K**) OCTA images of the SCP obtained after treatment. The retinal vascular area is 4.30 mm^2^. (**L**) The enface image. (**M**) The corresponding OCT cross-sectional image. (**F**) OCTA images of the DCP obtained after treatment. The retinal vascular area is 4.39 mm^2^. (**G**) The enface image. (**H**) The corresponding OCT cross-sectional image. The patient shows a relatively preserved vascular area with favorable post-treatment visual acuity after intravitreal aflibercept treatment. SCP, superficial capillary plexus; DCP, deep capillary plexus.

**Figure 2:**
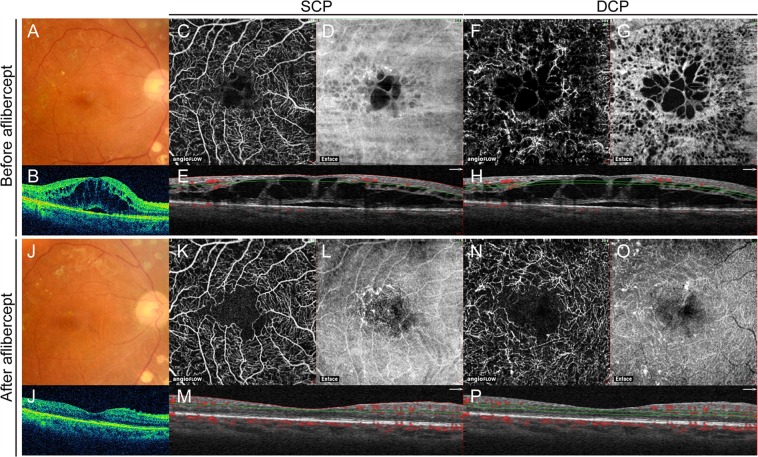
Retinal images obtained from a 70-year-old man before and after intravitreal aflibercept therapy for diabetic macular edema (DME). (**A**) Fundus photograph obtained before treatment. Visual acuity was 20/100. (**B**) Horizontally oriented spectral-domain optical coherence tomography (SD-OCT) images before treatment. Macular edema involving the fovea is apparent and central retinal thickness (CRT) is 683 µm. (**C**) Optical coherence tomography angiography (OCTA) images of the superficial capillary plexus (SCP) obtained before treatment. (**D**) The enface image. (**E**) The corresponding OCT cross-sectional image through the fovea is shown directly below. The retinal vascular area is 3.75 mm^2^. (**F**) OCTA images of the deep DCP obtained before treatment. (**G**) The enface image. (**H**) The corresponding OCT cross-sectional image through the fovea is shown directly below. The retinal vascular area is 3.61 mm^2^. (**I**) Fundus photograph obtained 5 months after treatment. The patient received three intravitreal aflibercept injections. Visual acuity was 20/40. (**J**) Horizontally oriented SD-OCT image after treatment. Macular edema is resolved and CRT is 227 µm. (**K**) OCTA images of the SCP obtained after treatment. The retinal vascular area is 3.75 mm^2^. (**L**) The enface image. (**M**) The corresponding OCT cross-sectional image. (**F**) OCTA images of the DCP obtained after treatment. The retinal vascular area is 3.66 mm^2^. (**G**) The enface image. (**H**) The corresponding OCT cross-sectional image. The patient shows a lower vascular area with worse visual acuity after intravitreal aflibercept compared with the eyes in Fig. 1. SCP, superficial capillary plexus; DCP, deep capillary plexus.

Table 2 shows OCTA findings within a 9 (3 × 3) mm^2^ area before and after intravitreal aflibercept injections. The mean retinal vascular area and the FAZ did not significantly change before and after intravitreal aflibercept treatment in both the SCP and the DCP (Table 2, Figs 1 and 2). The pre-treatment and post-treatment retinal vascular area in the SCP was 3.82 ± 0.38 (range: 2.84–4.46) and 3.82 ± 0.37 (2.98–4.46), respectively (P = 0.994). The pre-treatment and post-treatment retinal vascular area in the DCP was 3.88 ± 0.55 (2.46–4.66) and 3.90 ± 0.62 (2.07–4.75), respectively (P = 0.842). The pre-treatment and post-treatment FAZ area in the SCP was 0.41 ± 0.20 (0.18–0.69) and 0.48 ± 0.24 (0.17–0.79), respectively (P = 0.112). The pre-treatment and post-treatment FAZ area in the DCP was 0.75 ± 0.34 (0.29–1.67) and 0.71 ± 0.33 (0.36–1.77), respectively (P = 0.192). The presence of MAs in the DCP was observed in 20 eyes (87%) before treatment and 13 eyes (57%) after treatment (P = 0.049).

**Table 2:** Optical coherence tomography angiography findings before and after intravitreal aflibercept treatment for diabetic macular edema.

	Pre-treatment	Post-treatment	P value
BCVA before treatment			
Landolt C acuity chart (mean; range)	0.52 (0.15–1.0)	0.71 (0.2–1.2)	
LogMAR (mean ± SD)	0.28 ± 0.23	0.15 ± 0.22	0.003
CRT (µm) (mean ± SD; range)	484 ± 133 (313–828)	275 ± 62 (190–458)	<0.001
Microaneurysm, no. (%)			
SCP	4 (17)	2 (9)	0.665
DCP	20 (87)	13 (57)	0.049
Retinal vascular area within 3 × 3 mm^2^ (%)			
SCP (mean ± SD; range)	3.82 ± 0.38 (2.84–4.46)	3.82 ± 0.37 (2.98–4.46)	0.994
DCP (mean ± SD; range)	3.88 ± 0.55 (2.46–4.66)	3.90 ± 0.62 (2.07–4.75)	0.842
FAZ area within 3 × 3 mm^2^ (mm^2^)			
SCP (mean ± SD; range)	0.41 ± 0.20 (0.18–0.69)	0.48 ± 0.24 (0.17–0.79)	0.112
DCP (mean ± SD; range)	0.75 ± 0.34 (0.29–1.67)	0.71 ± 0.33 (0.36–1.77)	0.192

BCVA, best-corrected visual acuity; logMAR, logarithm of the minimum angle of resolution; CRT, central retinal thickness; SCP, superficial capillary plexus; DCP, deep capillary plexus; FAZ, foveal avascular zone; SD, standard deviation.

### Association Between Pre-Treatment VA and Retinal Microvasculature

Based on the univariate regression analysis, better BCVA at baseline before intravitreal aflibercept injections was significantly associated with a larger pre-treatment retinal vascular area in the SCP (R^2^ = 0.294, P = 0.008) and the DCP (R^2^ = 0.264, P = 0.012) although there was no association with the pre-treatment FAZ area in the SCP and the DCP (R^2^ = 0.038, P = 0.375 and R^2^ = 0.092, P = 0.159, respectively). The better retinal vascular area in both SCP and DCP was significantly associated with NPDR but not PDR (R^2^ = 0.243, P = 0.017 and R^2^ = 0.179, P = 0.044, respectively) and less macular edema (CRT) before treatment (R^2^ = 0.253, P = 0.014 and R^2^ = 0.179, P = 0.044, respectively).

### Association Between Post-Treatment VA and Retinal Microvasculature

Better BCVA after intravitreal aflibercept treatment was significantly associated with better pre-treatment logMAR BCVA (R^2^ = 0.452, P < 0.001), NPDR but not PDR (R^2^ = 0.394, P = 0.001), better pre-treatment retinal vascular area in the SCP (R^2^ = 0.512, P < 0.001) and the DCP (R^2^ = 0.361, P = 0.002), better post-treatment retinal vascular area in the SCP (R^2^ = 0.717, P < 0.001) and the DCP ((R^2^ = 0.618, P < 0.001), and the smaller post-treatment FAZ in the DCP (R^2^ = 0.274, P = 0.010) (Table 3).

**Table 3:** Optical coherence tomography angiography parameters associated with post-treatment best-corrected visual acuity after intravitreal aflibercept.

Variable	Univariate linear regression		
	Regression coefficient	R^2^	P value
Age	−0.0014	0.006	0.732
Sex (male: 0, female: 1)	−0.179	0.138	0.081
NPDR or PDR (NPDR:0, PDR:1)	0.272	0.394	0.001
BCVA before treatment	0.625	0.452	<0.001
No. of injections	0.066	0.16	0.059
Retinal vascular area before treatment			
SCP	−0.409	0.512	<0.001
DCP	−0.236	0.361	0.002
FAZ (mm^2^) before treatment			
SCP	0.187	0.029	0.438
DCP	0.257	0.16	0.058
FAZ (mm^2^) after treatment			
SCP	0.266	0.084	0.179
DCP	0.345	0.274	0.01
Retinal vascular area after treatment			
SCP	−0.489	0.717	<0.001
DCP	−0.274	0.618	<0.001

BCVA, best-corrected visual acuity; logMAR, logarithm of the minimum angle of resolution; CRT, central retinal thickness; NPDR, nonproliferative diabetic retinopathy; PDR, proliferative diabetic retinopathy; SCP, superficial capillary plexus; DCP, deep capillary plexus; FAZ, foveal avascular zone; SD, standard deviation.

### Resolution of DME

**Figure 3:**
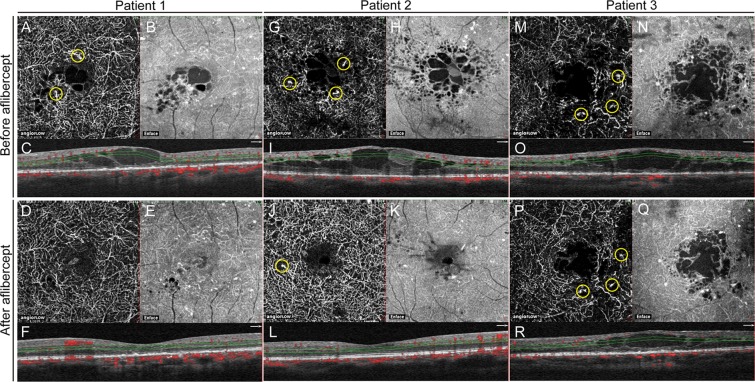
Representative images of the deep capillary plexus (DCP) obtained from a 57-year-old man (patient 1; resolved diabetic macular edema), a 48-year-old man (patient 2; resolved diabetic macular edema), and a 55-year-old man (patient 3; persistent diabetic macular edema). (**A**) Optical coherence tomography angiography (OCTA) image of the DCP before treatment in patient 1. Yellow circles indicate microaneurysms (MAs). Visual acuity was 20/22. (**B**) The enface image showing the area of macular edema. (**C**) The corresponding OCT cross-sectional image through the fovea is shown directly below. Macular edema involving the fovea is apparent and central retinal thickness (CRT) is 543 µm. The retinal vascular area is 4.02 mm^2^. (**D**) OCTA image of the DCP 8 months after treatment. The patient received one intravitreal aflibercept injection. The MAs disappeared. Visual acuity was 20/20. (**E**) The enface image. (**F**) The corresponding OCT cross-sectional image. Macular edema involving the fovea is resolved and CRT is 263 µm. The retinal vascular area is 4.19 mm^2^. (**G**) OCTA image of the DCP before treatment in patient 2. Yellow circles indicate microaneurysms (MAs). Visual acuity was 20/29. (**H**) The enface image showing the area of macular edema. (**I**) The corresponding OCT cross-sectional image through the fovea is shown directly below. Macular edema involving the fovea is apparent and CRT is 553 µm. The retinal vascular area is 4.26 mm^2^. (**J**) OCTA image of the DCP 7 months after treatment. The patient received two intravitreal aflibercept injections. The MAs are reduced but remain (yellow circle). Visual acuity was 20/20. (**K**) The enface image. (**L**) The corresponding OCT cross-sectional image. Macular edema involving the fovea is resolved and CRT is 289 µm. The retinal vascular area was 4.64 mm^2^. (**M**) OCTA image of the DCP before treatment in patient 3. Yellow circles indicate MAs. Visual acuity was 20/100. (**N**) The enface image showing the area of macular edema. (**O**) The corresponding OCT cross-sectional image through the fovea is shown directly below. Macular edema involving the fovea is apparent and CRT is 640 µm. The perfused area is 2.87 mm^2^. (**P**) OCTA image of the DCP 4 months after treatment. The patient received three intravitreal aflibercept injections. The MAs remain (yellow circle). Visual acuity was 20/100. (**Q**) The enface image. (**R**) The corresponding OCT cross-sectional image. Macular edema involving the fovea persists and CRT is 383 µm. The perfused area is 3.12 mm^2^.

Resolution of the DME (CRT of 300 µm or less after final intravitreal aflibercept) was observed in 17 eyes (74%), whereas persistence of DME (CRT over 300 µm after final intravitreal aflibercept) was identified in six eyes (26%) (Table 4). The mean CRT was 249 ± 38 µm in resolved DME and 348 ± 61 µm in persistent DME after treatment (P < 0.001). Although most eyes in both groups had MAs in the DCP before treatment (82% in resolved DME versus 100% in persistent DME), the number of eyes with MAs in the DCP were significantly higher in the persistent DME group (100%) than in the resolved DME group (41%) after treatment (P = 0.019) (Fig. 3). The visual gain was significantly worse in the persistent DME group after treatment (P = 0.031).

**Table 4:** Comparisons of Optical Coherence Tomography Angiography Parameters in Resolved and Persistent DME after intravitreal aflibercept.

	Resolved DME (n = 17)	Persistent DME (n = 6)	P value
CRT before treatment (µm) (mean ± SD)	471 ± 118	489 ± 141	0.778
Microaneurysm before treatment, no. (%)			
SCP	3 (18)	1 (17)	1
DCP	14 (82)	6 (100)	0.539
Vessel area within 3 × 3 mm^2^ before treatment (mm^2^)			
SCP (mean ± SD)	3.802 ± 0.393	3.868 ± 0.358	0.723
DCP (mean ± SD)	3.890 ± 0.572	3.859 ± 0.529	0.911
FAZ area within 3 × 3 mm^2^ before treatment (mm^2^)			
SCP (mean ± SD)	0.442 ± 0.193	0.331 ± 0.197	0.244
DCP (mean ± SD)	0.757 ± 0.354	0.735 ± 0.311	0.894
CRT after treatment (µm) (mean ± SD)	249 ± 38	348 ± 61	<0.001
Microaneurysm after treatment, no. (%)			
SCP	1 (6)	1 (17)	0.462
DCP	7 (41)	6 (100)	0.019
Vessel area within 3 × 3 mm^2^ after treatment (mm^2^)			
SCP (mean ± SD; range)	3.826 ± 0.372	3.799 ± 0.413	0.883
DCP (mean ± SD; range)	3.902 ± 0.647	3.887 ± 0.585	0.961
FAZ area within 3 × 3 mm after treatment (mm^2^)			
SCP (mean ± SD; range)	0.512 ± 0.242	0.371 ± 0.195	0.216
DCP (mean ± SD; range)	0.690 ± 0.348	0.755 ± 0.285	0.684
LogMAR BCVA after treatment (mean ± SD)	0.132 ± 0.208	0.210 ± 0.247	0.501
Visual gain*	+0.169 ± 0.188	+0.008 ± 0.09	0.031
Number of injections (mean ± SD)	2.6 ± 1.2	2.7 ± 1.8	0.774

BCVA, best-corrected visual acuity; logMAR, logarithm of the minimum angle of resolution; CRT, central retinal thickness; NPDR, nonproliferative diabetic retinopathy; PDR, proliferative diabetic retinopathy; SCP, superficial capillary plexus; DCP, deep capillary plexus; FAZ, foveal avascular zone; SD, standard deviation.

*The visual gain is the difference between LogMAR BCVA after treatment and before treatment.

Resolved or persistent DME was defined as having a CRT below 300 µm or over 300 µm, respectively, after intravitreal aflibercept injection.

## Discussion

Our results reveal that both retinal vascular area and the FAZ within the 9 (3 × 3) mm^2^ area did not significantly differ before and after intravitreal aflibercept treatment in patients with DME. That is, repeated administration of intravitreal aflibercept with a mean of 2.6 injections over the course of 8.5 months maintained the retinal perfusion at the macula in eyes with DME. This result is consistent with previous studies showing no significant change in vessel density and the FAZ area 1 month after anti-VEGF treatment^25^. Since we could not evaluate potential changes in retinal vascular area and the FAZ in eyes without treatment, it remains unclear whether intravitreal aflibercept has a preventative effect on the progression of macular ischemia. However, the current study suggests that intravitreal aflibercept injections following a PRN regimen may at least not lead to a deterioration of retinal perfusion in most eyes with DME.

Before treatment with intravitreal aflibercept, worse BCVA was significantly associated with less retinal vascular area in the SCP and the DCP. In addition, less retinal vascular area in the SCP and the DCP was significantly correlated with the severity of the DR and the macular edema. High intraocular concentration of VEGF is associated not only with the macular edema but also with the progression of DR, including the progression of ischemia^26,27^. VEGF overexpression contributes to the increase in permeability as well as to the increase in the intercellular adhesion molecule-1 (ICAM-1) expression in retinal vascular endothelium, and promotes ICAM-1-mediated leukocytes binding to the retinal vasculature, resulting in downstream non-perfusion^28–30^. Therefore, we speculate that macular edema and macular ischemia may thus progress concomitantly in eyes with DME, resulting in visual impairments as shown in the current study. Therefore, early initiation of the anti-VEGF therapy may help the prevention of the concomitant progression of macular edema and capillary ischemia^31^.

In the current study, BCVA significantly improved after intravitreal aflibercept administration, as expected. Better BCVA after intravitreal aflibercept administration was significantly associated with a larger retinal vascular area in the SCP and the DCP before treatment, indicating that baseline retinal vascular area not only correlates with baseline BCVA but may also be useful to predict visual outcomes after intravitreal aflibercept treatment. In addition, because better BCVA after intravitreal aflibercept treatment was also significantly associated with a larger retinal vascular area in the SCP and the DCP and a smaller FAZ area in the DCP after treatment, the preservation of retinal perfusion after treatment seems to be crucial for better visual outcomes. Therefore, strategies that maintain or increase retinal perfusion are expected to further improve visual outcomes following intravitreal aflibercept administration in eyes with DME.

It has been reported that MAs are seen more frequently in the DCP than in the SCP and are associated with the development of DME^17,32–35^. Here, consistent with previous studies, MAs were observed in the DCP in 20 eyes (87%), although they were only detected in four eyes (17%) in the SCP before treatment. The number of eyes with MAs in the DCP significantly decreased to 13 eyes (P = 0.049), indicating that the anti-VEGF therapy resolved the MA formation in some eyes. Because increased VEGF expression has been shown to play a significant role in MA formation by inducing focal endothelial proliferation^36^, it is reasonable to assume that the inhibition of VEGF may suppress MAs^27^. However, more than half of the patients had persistent MAs, even after anti-VEGF treatment, that were associated with persistent DME. In our case series, MAs in the DCP were identified in all six eyes with persistent DME, even after repeated aflibercept administration. In contrast, a significantly lower number of eyes (seven of 17 eyes; 41%) had MAs in the DCP in resolved DME (P = 0.019). The visual gain was significantly worse in persistent DME than in resolved DME (P = 0.031). Therefore, we speculate that a smaller effect of VEGF inhibition on MA regression may be responsible for persistent DME and poor visual improvement. Further studies are required to elucidate the differences in MA responses and strategies for the treatment of persistent DME associated with non-resolved MA.

This study has several limitations, including its retrospective design, the variable follow-up period, and a relatively small sample size. We could not evaluate vessel diameter. Furthermore, we only analyzed a limited area (3 × 3 mm^2^) of retinal microvasculature, which is important for central vision but may not reflect the entire disease process in DME. The inability to fully eliminate autosegmentation errors may also have influenced the measurements of microaneurysms and retinal vascular area especially in the DCP. Therefore, further studies with large number of patients are necessary to validate current results and to improve understanding of microvascular changes before and after intravitreal aflibercept in DME. Nevertheless, the current study reveals that vascular perfusion in both the SCP and the DCP is maintained after intravitreal aflibercept administration, and that preservation of retinal vascular area seems crucial for better visual outcomes. In addition, we identified a significant association between persistent MA formation and persistent DME, responsible for lower visual gain after intravitreal aflibercept treatment. These findings are valuable for our understanding of the changes in microvasculature associated with diabetes and for the improvement of the management of DME.

In conclusion, our study shoewed that the retinal vascular area and the FAZ were maintained after intravitreal aflibercept administration in patients with DME. A larger retinal vascular area and a smaller FAZ area were associated with better visual acuity after treatment. Persistent MAs were associated with persistent DME and poor visual improvement. Further studies are needed to develop strategies to maintain or increase retinal blood flow and resolve MAs, for better long-term visual prognosis in eyes with DME.

## Methods

This study represents a retrospective consecutive case series. All procedures were in adherence with the tenets of the declaration of Helsinki, and were approved by the institutional review board of Osaka University Graduate School of Medicine (10039). Patients with macular edema secondary to diabetic retinopathy who had been treated with intravitreal aflibercept between December 2014 and February 2016 at the Department of Ophthalmology, Osaka University Hospital, were enrolled in this study. All patients provided written informed consent before intravitreal aflibercept. Written informed consent was not considered to be necessary by the Ethics Committee to participate in this retrospective study.

### OCTA Examination

OCTA was performed using an AngioVue instrument (Optovue RTVue XR Avanti; Optovue, Inc., Fremont, CA, USA). Automated segmentation using the AngioVue module was performed on the same day as the clinical examination. In each eye, a 3 × 3-mm^2^ area centered on the fovea was scanned. The retinal vascular area was automatically segmented into four layers: the superficial capillary plexus (SCP), which includes vasculature from the internal limiting membrane to 15 μm beneath the inner plexiform layer; the deep capillary plexus (DCP), which includes vasculature from 15 to 70 μm beneath the inner plexiform layer; the outer retina; and the choroid. We quantitatively assessed the retinal vascular area of the SCP and the DCP in the fovea using the AngioVue instrument. The retinal vascular area was defined as the percentage of the entire area occupied by large vessels and microvasculature in a particular region, and was calculated within the 3 × 3-mm^2^ scan area in each eye. The FAZ area was also measured within the 3 × 3-mm^2^ area in the SCP and the DCP. Microaneurysms was defined as a saccular enlargement of the retinal capillaries. The number of microaneurysms in the SCP and the DCP was examined by two masked investigators (CB, AW). In the event of disagreement, a third investigator (TW) was consulted to reach a consensus. OCTA images with poor quality (signal strength index ≤ 50), motion artifacts, or incorrect autosegmentation were excluded from all analyses. The segmentation error with irregularly wavy border was excluded by two masked investigators (CB, AW) and a third investigator (TW) was consulted to derive a final determination in the event of disagreement.

### SD-OCT Examination

Patients were examined with a Cirrus SD-OCT (Carl Zeiss Meditec, Inc., Dublin, CA, USA) before and after intravitreal aflibercept injections. Central retinal thickness measurements were acquired by measurements within a 1-mm diameter of the fovea.

### Intravitreal Aflibercept

All patients were examined monthly at the Osaka University hospital and received treatment with intravitreal aflibercept. The patients received one to three initial intravitreal anti-VEGF injections decided by retina specialists, followed by a pro re nata (PRN) regimen with monthly monitoring. Retreatment with intravitreal aflibercept was considered if a patient showed macular edema on SD-OCT imaging with a central retinal thickness (CRT) of more than 300 µm with intraretinal or subretinal exudation.

### Data Collection and Statistical Analyses

The following data were collected: ophthalmic history, baseline best corrected visual acuity (BCVA), baseline CRT, baseline OCTA images, post-treatment BCVA, post-treatment CRT, post-treatment OCTA images. Post-treatment examination was conducted at final follow-up visit in each patient. The main outcome measures were post-treatment BCVA and OCTA parameters in the SCP and the DCP. All pre-treatment and post-treatment data were collected on the same day before and after treatment, respectively. For statistical analysis, the BCVA was measured using the Landolt C acuity chart and analyzed on a logarithm of the minimum angle of resolution (logMAR) scale. Univariate regression analysis was performed to investigate the associations between logMAR BCVA and OCTA parameters for the vascular area and the FAZ area. All analyses were conducted using SigmaStat software version 3.1 (SPSS Inc., Chicago, IL, USA) and JMP Pro Software (SAS Inc., Cary, NC, USA). *P* < 0.05 indicated statistical significance.
